# Is Preemptive Kidney Transplantation Associated With Improved Outcomes when Compared to Non-preemptive Kidney Transplantation in Children? A Systematic Review and Meta-Analysis

**DOI:** 10.3389/ti.2022.10315

**Published:** 2022-03-17

**Authors:** Reshma Rana Magar, Simon Knight, Jelena Stojanovic, Stephen D. Marks, Jeffrey A. Lafranca, Samuel Turner, Frank J. M. F. Dor, Liset H. M. Pengel

**Affiliations:** ^1^ Centre for Evidence in Transplantation, Nuffield Department of Surgical Sciences, University of Oxford, Oxford, United Kingdom; ^2^ Great Ormond Street Hospital for Children, London, United Kingdom; ^3^ Department of Paediatric Nephrology, Great Ormond Street Hospital for Children NHS Foundation Trust, London, United Kingdom; ^4^ NIHR Great Ormond Street Hospital Biomedical Research Centre, University College London Great Ormond Street Institute of Child Health, London, United Kingdom; ^5^ Imperial College Renal and Transplant Centre, Hammersmith Hospital, London, United Kingdom; ^6^ North Bristol NHS Trust, Bristol, United Kingdom

**Keywords:** outcomes, meta-analysis, systematic review, paediatric, preemptive kidney transplantation

## Abstract

**Main Problem:** Preemptive kidney transplantation (PKT) is performed prior to dialysis initiation to avoid dialysis-related morbidity and mortality in children and adolescents. We undertook a systematic review to compare clinical outcomes in PKT versus kidney transplantation after dialysis initiation in paediatric patients.

**Methods:** The bibliographic search identified studies that compared paediatric recipients of a first or subsequent, living or deceased donor PKT versus non-preemptive kidney transplant. Methodological quality was assessed for all studies. Data were pooled using the random-effects model.

**Results:** Twenty-two studies (*n* = 22,622) were included. PKT reduced the risk of overall graft loss (relative risk (RR) .57, 95% CI: .49–.66) and acute rejection (RR: .81, 95% CI: .75–.88) compared to transplantation after dialysis. Although no significant difference was observed in overall patient mortality, the risk of patient death was found to be significantly lower in PKT patients with living donor transplants (RR: .53, 95% CI: .34–.83). No significant difference was observed in the incidence of delayed graft function.

**Conclusion:** Evidence from observational studies suggests that PKT is associated with a reduction in the risk of acute rejection and graft loss. Efforts should be made to promote and improve rates of PKT in this group of patients (PROSPERO).

**Systematic Review Registration:** https://clinicaltrials.gov/, CRD42014010565

## Introduction

Kidney transplantation (KT) is the treatment of choice for children with end-stage kidney disease (ESKD) as it offers better survival and quality of life compared to treatment with dialysis (1, 2). Preemptive kidney transplantation (PKT) is performed before the initiation of dialysis to avoid the morbidity and mortality associated with dialysis (3, 4). Whether or not PKT also leads to improved clinical outcomes has been addressed by several studies but these report mixed findings. A USA registry analysis showed significantly better 5-year patient and graft survival rates in children transplanted preemptively vs. non-preemptively (nPKT) (5), whilst a multicentre retrospective cohort study from Japan found no difference in either patient survival or 5-year graft survival between these groups (6). Likewise, a number of single centre studies also show inconsistent results (7–10).

Historically, some centres believed that children with chronic kidney disease had to progress to ESKD requiring dialysis before being offered KT. The experience of dialysis would give children a sense of what life was like on dialysis leading to improved adherence post-transplant (11). This practice is no longer supported in most paediatric nephrology centres.

Paediatric ESKD patients differ from adult patients in terms of causes of ESKD, donor-recipient size mismatch, post-transplant complications, medication non-adherence, growth and development complications, and co-morbidities associated with the lower urinary tract (12). Therefore, it is important to evaluate the benefits of PKT specifically for the paediatric population. We undertook a systematic review to determine whether it is beneficial for paediatric patients to undergo KT before dialysis is initiated.

## Materials and Methods

### Registration of Protocol

This study was designed and reported according to the PRISMA guidelines (13). The protocol was prospectively registered with PROSPERO (CRD42014010565) (14).

### Inclusion Criteria

Type of studies: Any study design, including registry analyses, cohort studies, case-control studies and case series comparing PKT with nPKT, were eligible for inclusion. Case reports, and narrative reviews, editorials without primary data and non-English studies were excluded. We included both full articles and congress abstracts, and also checked for overlap in case abstracts were later published as full texts.

Type of participants and intervention: Eligible studies included those that compared paediatric recipients of a first or subsequent, living donor (LD) or deceased donor (DD) PKT versus nPKT. We included studies that described their population as paediatric or reported an age range of up to 18 years. PKT was defined as transplantation prior to any initiation of peritoneal dialysis (PD) or haemodialysis (HD). nPKT refers to transplantation after any given period of PD or HD. No restrictions were imposed on pre-transplant dialysis duration (dialysis vintage). Studies reporting on recipients with either a history of a previous organ transplant other than kidney or recipients of multi-organ transplants were excluded.

Type of outcomes: The outcomes of interest were overall graft loss (non-censored for death), death-censored graft loss, patient death (from all causes), delayed graft function (DGF), incidence of acute rejection (any definition, including clinically suspected and biopsy-proven acute rejection), renal function [serum creatinine or estimated glomerular filtration rate (eGFR)], primary non-function, quality of life, return to school after transplantation, height/growth measures, and incidence of cardiovascular morbidity, infections and malignancy.

### Search Strategy

As this review was part of a larger study that reviewed the available evidence for both paediatric and adult KT patients, a broad bibliographic search was carried out up to 31 July 2020 using a mixture of free text and controlled vocabulary terms (Supplementary Table S1), which retrieved references for both paediatric and adult studies. Five electronic databases including EMBASE, MEDLINE (OvidSP), Cochrane Central Register of Controlled Trials (CENTRAL), Web-of-science and Google Scholar were searched. No limits for date of publication or language were applied. The references of identified studies or review articles were scanned to find potentially eligible studies that may have been missed during the literature search. Attempts were made to contact the study authors in case of missing data or unclear study information.

### Selection of Studies

The study selection was carried out in two stages by independent reviewers (RRM, LP, ST, and JL). Initially, titles and abstracts of the retrieved studies were screened against the inclusion/exclusion criteria, followed by full-text review of potentially eligible papers and final selection of the studies to be included in the review. Discrepancies between reviewers were resolved by consensus.

### Data Extraction

Two reviewers (RRM and LP) independently extracted the data using a standardized data extraction sheet. Discrepancies between reviewers were solved by discussion. Where there was more than one publication of the same study, data were only extracted from the publication that had the most complete data or the largest sample size. We extracted data on general study information and demographics, and primary and secondary outcomes. Where possible, data for LD and DD were extracted separately.

### Assessment of Methodological Quality

The methodological quality of the included studies, published as full text papers, was assessed by two independent authors (RRM and LP) using the Downs and Black checklist (15). Two out of the 27 items from the checklist were removed, i.e., the items relating to intervention compliance and the power of the study, as these were considered irrelevant or could not be calculated.

### Statistical Analysis

Where at least three studies reported on an outcome, meta-analysis was performed using the statistical software R version 3.6.3. Data were pooled using the random-effects model to calculate the relative risk (RR) with a 95% confidence interval (CI). We planned to analyze data according to LD vs. DD, however, this was not always feasible as most studies combined LD and DD in their analyses. Hence, data were pooled regardless of whether they were LD and/or DD. Patient or graft survival rates were converted to the number of deaths and graft losses. Data on graft loss were categorized as either overall graft loss or death-censored graft loss. If a study neither defined graft loss nor specified whether the graft loss data was death-censored or non-censored for death, we categorized graft loss as being non-censored for death. We calculated a pooled estimate for the nPKT group if the study reported the results for nPKT according to different dialysis durations or separately for PD or HD. If a single study reported an outcome at more than one time point, the most recent follow-up data was used. Data were pooled for any duration of follow up. In order to account for the role of confounders in the analysis of the overall graft loss, we also calculated a pooled ratio consisting of adjusted ratios either calculated or directly extracted from the studies. Secondary analyses were conducted excluding smaller studies with overlapping countries and study periods to avoid duplicate use of data. If less than three studies reported on an outcome we summarized the results in a narrative review.

Heterogeneity was analyzed using the I^2^ statistic (16). Where heterogeneity was significant (I^2^ ≥ 50%), a mixed effect analysis was performed to explore its potential causes.

## Results

### Included Studies

**FIGURE 1:**
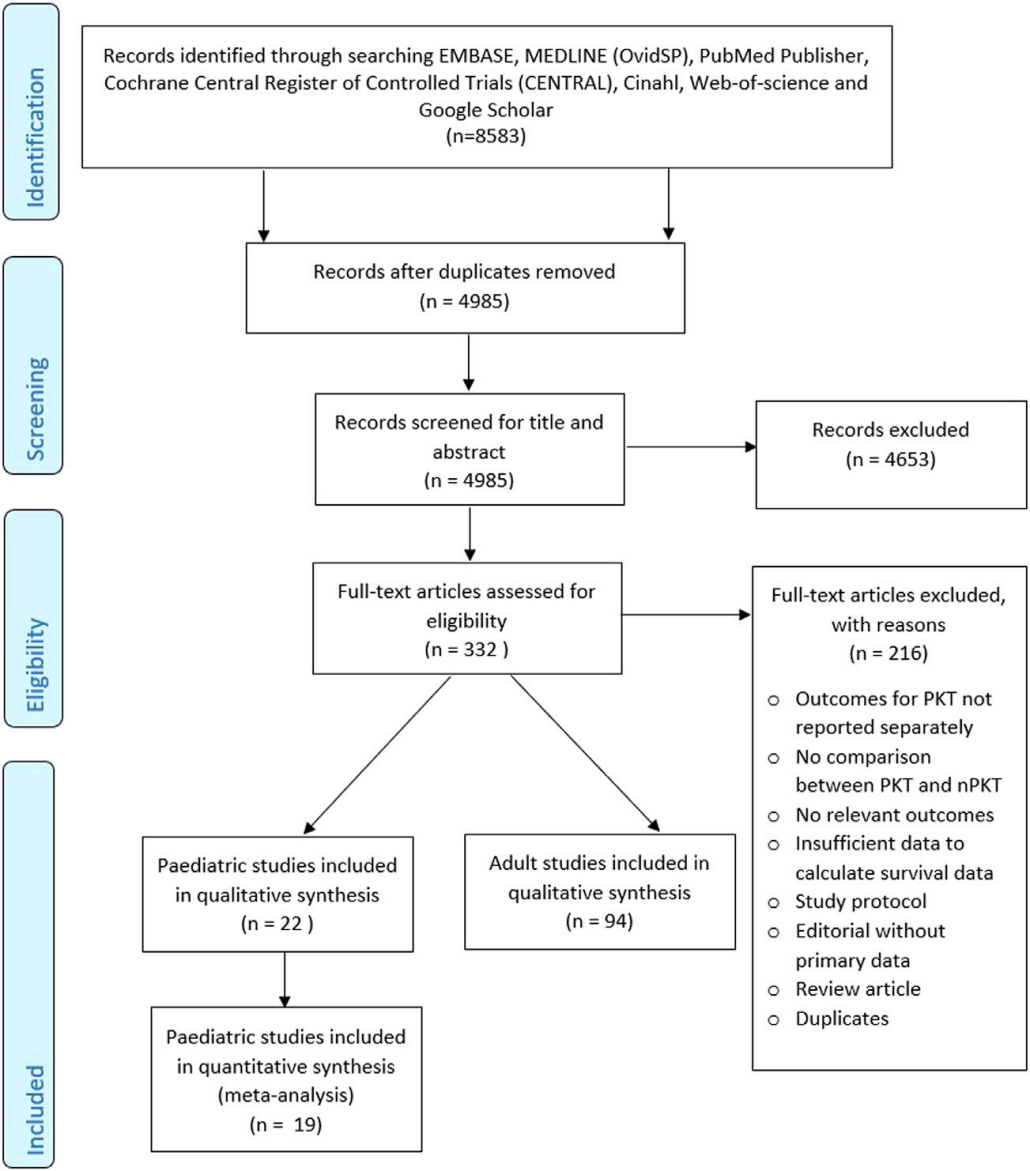
PRISMA flow diagram.

The literature search retrieved 8,583 references. Following full-text analysis of 332 studies, 216 studies were excluded (Figure 1). Of the remaining 116 studies that met the inclusion criteria, 22 were identified as paediatric studies reporting on a total of 22,622 patients (Table 1). Cransberg (17) and Cransberg (18) were considered as one study due to insufficient data on the extent of overlap between the studies. Only the estimate for adjusted graft survival was extracted from Cransberg (18).

**TABLE 1:** Characteristics of the included studies.

Author (year); country	Study design and setting	Paediatric definition	1st Tx only	Number of patients					% Of HD in nPKT	HLA mismatch (Mean ± SD)		Duration of follow up	
	Period when Tx was received			LD		DD		Total					
				PKT	nPKT	PKT	nPKT			PKT	nPKT	PKT	nPKT
Amaral (5) (2016); United States	Retrospective registry analysis; multicentre	<18 y	Yes	1,104	2,266	564	3,593	7,527	NR	3.26	3.79	NR	NR
	January 2000–September 2012												
Atkinson (24) (2020); United States	Prospective cohort study; multicentre	<17 y	Yes	50	41	29	50	170	41.7	—	—	Median: 3.8 yIQR: 1.8–5.8 y	NR
	March 2006–January 2017												
Butani (25) (2011); United States	Retrospective registry analysis; multicentre	<17 y	Yes	730	1,354	273	1,249	3,606	47.6	2.8 ± 0*	—	5 y	5 y
	January 1995–December 2000												
Cransberg (17) (2006); Europe	Retrospective registry analysis; multicentre	<16 y	Yes	86	132	70	825	1,113	NR	2.3 (LD); 2.6 (DD)	2.1 (LD); 2.5 (DD)	Mean = Median = 5.3 y	Mean = Median = 5.3 y
Cransberg (18) (2000); Netherlands	January 1990–January 2000											Range: 0–14.1 y	Range: 0–14.1 y
Cuervo (19) (2007); Mexico	Cohort study; single centre	NR	NR	17	13	2	6	38	NR	—	—	NR	NR
	January 1995–December 2003												
Duzova (32) (2009); Turkey	Retrospective cohort studies; single centre	NR	NR	13	17	4	12	46	NR	—	—	5 y	5 y
	2000–2008												
Fitzwater (30) (1991); United States	Retrospective cohort studies; single centre	<18 y	Yes	13	17	0	16	46	75.8	—	—	Mean: 24 m	Mean ± SD: 19.5 ± 7 m
	Until 1987												
Flom (26) (1992); United States	Retrospective cohort studies; single centre	NR	No	26	40	0	0	66	32.5	—	—	Median: 3.5 yRange: 0.5–7.1 y	Median: 4.35 yRange: 0.6–7.3 y
	January 1984–December 1990												
Garcia (9) (2015); Brazil	Retrospective cohort study; single centre	NR	NR	49	109	32	133	323	26.4	—	—	Median: 36 mIQR: 13–68 m	Median: 42 mIQR: 17–69 m
	January 2000–December 2010												
Harada (6) (2001); Japan	Retrospective cohort studies; single centre	≤18 y	NR	9	20	—	—	29	45.0	2.2 ± 0.70	2.3 ± 0.87	Mean ± SD: 42.4 ± 19.4 m	Mean ± SD: 68.3 ± 39.8 m
	August 1987–December 1998												
Kaya (20) (2018); Turkey	Retrospective cohort study; single centre2005–2017	NR	NR	—	—	—	—	230	NR	—	—	Median: 7.23 yMean ± SD: 4.71 ± 2.61 y	Median: 7.23 yMean ± SD: 5.88 ± 9.38 y
Kim (27) (2019); Canada	Retrospective cohort study; single centre	<18 y	No	54	98	21	151	324	51.0	—	—	1 y	1 y
	January 2000–December 2015												
Kramer (21) (2012); Europe	Retrospective registry analysis; multicentre	>3 and <18 y	Yes	321	435	123	950	1829	NR	—	—	8 y	8 y
	January 1988–December 2007												
Mahmoud (22) (1997); France	Retrospective cohort study; single centre	NR	NR	8	8	32	55	103	82.5	3.3	3.3	Mean: 3.3 y Range: 0.8–7.0 y	Mean: 3.2 yRange: 0.4–7.8 y
	April 1987–December 1994												
Marlais (28) (2018); United Kingdom	Retrospective registry analysis; multicentre	<18 y	NR	607	—	—	—	2038	44.9	—	—	NR	NR
	January 2000–December 2015												
Naderi (10), (2017); Iran	Retrospective cohort study; single centre	≤18 y	No	—	—	—	—	314	89.2	—	—	Mean ± SD: 15.9 ± 4.0 y	Mean ± SD: 15.9 ± 4.0 y
	1989 to 2013											Range: 0.5–20 y	Range: 0.5–20 y
Nevins (7) (1991); United States	Retrospective cohort study; single centre	<6 y	Yes	31	24	2	13	70	56.8	—	—	5 y	5 y
	July 1979–October 1987												
Offner (8) (1993); Germany	Retrospective cohort study; single centre	NR	Yes	14	14	14	14	56	NR	—	—	5 y	5 y
	January 1970–September 1991												
Reydit (29) (2017); France	Retrospective cohort study; multicentre	≤18 y	Yes	-	-	-	-	1920	NR	—	—	Median: 7 y	Median: 7 y
	1995–2013												
Sinha (31) (2010); United Kingdom	Cross-sectional study; single centre	NR	NR	16	46	23	44	129	42.2	1.83	2.14	Median: 4 yRange: 1–12 y	Median: 4 yRange:1–15 y
	May 1993–November 2006												
Splinter (33) (2018); Netherlands, Belgium and Germany	Cross-sectional study; multicentre	8–18 y	NR	-	-	-	-	150	NR	—	—	N/A	N/A
	October 2007–December 2014												
Vats (23) (2000); United States	Retrospective registry analysis; multicentre	NR	Yes	466	890	159	980	2,495	60.4	—	—	Mean ± SD: 28.6 ± 19.5 m	Mean ± SD: 27.3 ± 19.0 m
	1992–1996										

PKT, preemptive kidney transplantation; nPKT, non-preemptive kidney transplantation; HD, haemodialysis; Tx, transplant; DD, deceased donor; LD, living donor; *, standard error; SD, standard deviation; IQR, interquartile range; y, years; m, months; NR, not reported; N/A, not applicable.

### Methodological Quality

The methodological quality of the included studies varied with quality scores ranging from 10 to 19 out of a maximum possible score of 26 (Supplementary Table S2). Eleven studies adjusted for confounders in their analysis.

### Patient Death

**FIGURE 2:**
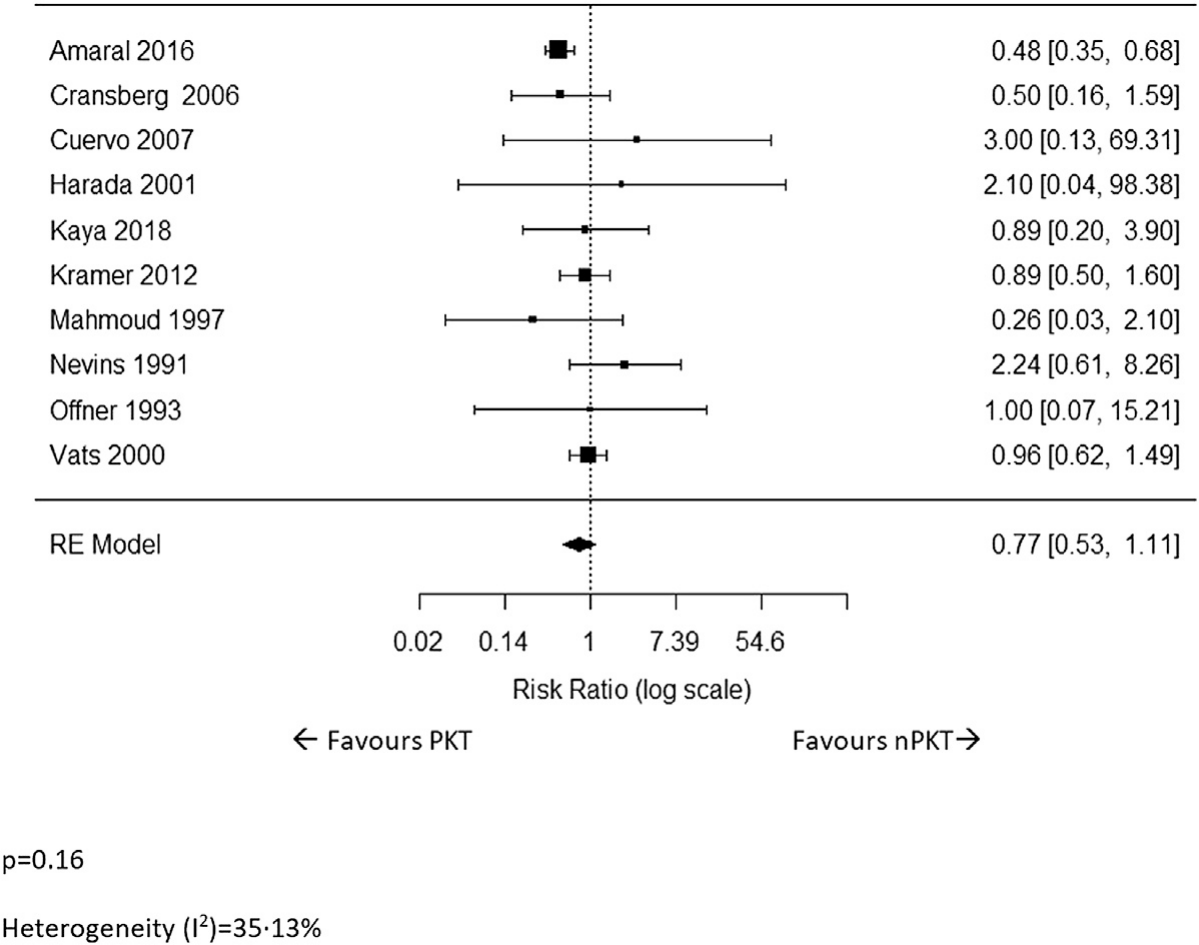
The relative risk of patient death for PKT vs. nPKT.

Ten studies (5–8, 17, 19–23) reported data on patient deaths. The pooled analysis showed no significant difference in the risk of patient death for PKT vs. nPKT (*n* = 13,490; RR: .77; CI: .53–1.11; *p* = .16; Figure 2). Heterogeneity was not significant (I^2^ = 35.13%). The difference in the risk remained nonsignificant after excluding four studies (8, 17, 20, 22) with overlapping countries and study periods (*n* = 11,988; RR: .86; CI: .53–1.39; *p* = .53; I^2^ = 57.94%; Supplementary Figure S1).

Patient death for LD transplants was reported in three studies (5, 6, 17). The pooled analysis revealed a significantly lower risk of patient death in PKT patients (*n* = 3,617; RR: .53; CI: .34–.83; *p* = .0054; I^2^ = 0%; Supplementary Figure S2).

Two studies (5, 17) reported data on patient survival for DD. Amaral et al (5) reported a significantly higher 5-year patient survival in the PKT versus nPKT group (97.5% vs. 95.0%; *p* = .004). However, in the Cransberg et al (17) study, patient survival at 6 years following transplantation was similar between these groups.

### Graft Loss

**FIGURE 3:**
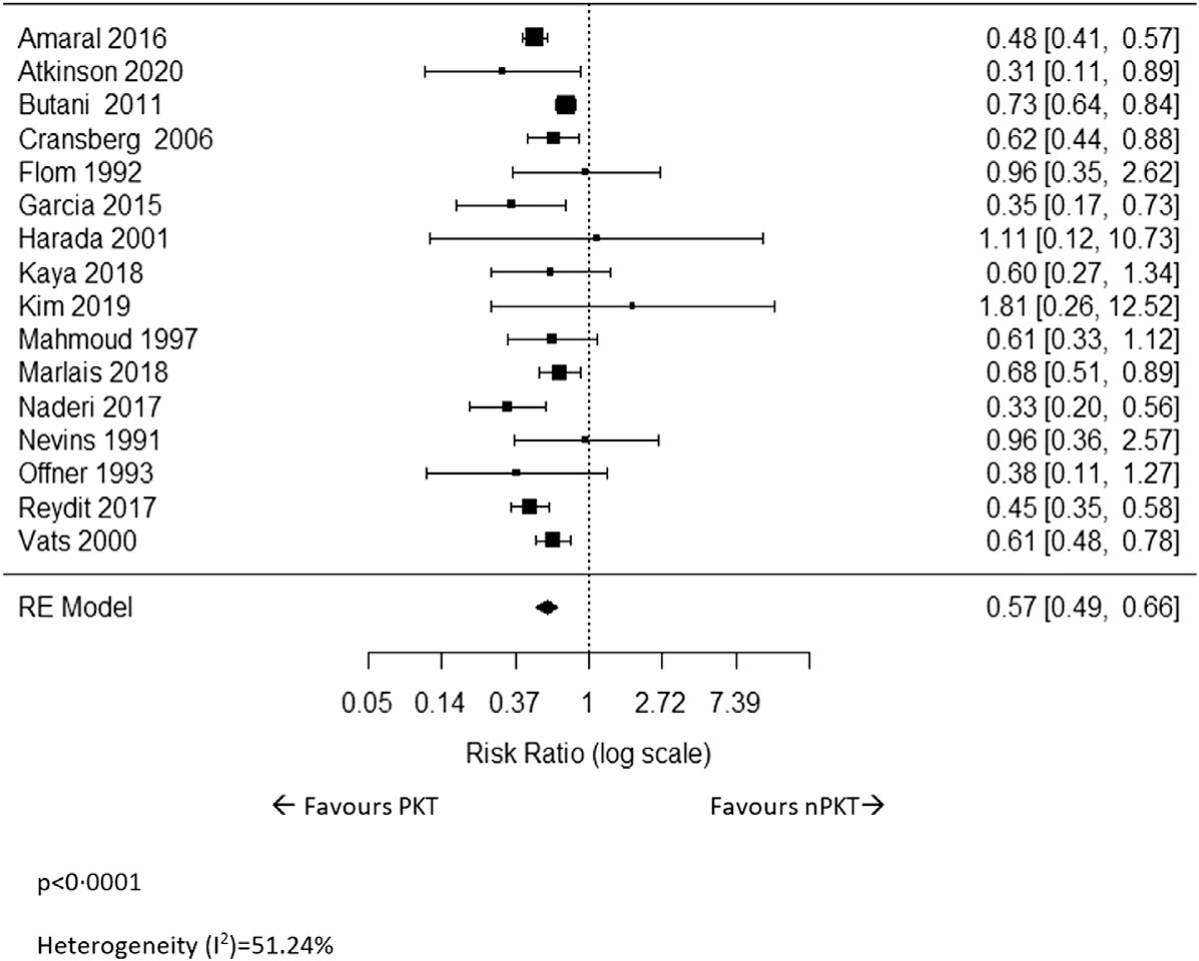
The relative risk of overall graft loss for PKT vs. nPKT.

Sixteen studies (5–10, 17, 20, 22–29) reported on overall graft loss. The meta-analysis revealed that the risk of graft loss following PKT was significantly lower than that of nPKT (*n* = 20,212; RR: .57; CI: .49–.66; *p* < .0001; I^2^ = 51.24%; Figure 3). Results were similar after excluding four (8, 24–26) studies with overlapping countries and study periods (*n* = 16,314; RR: .54; CI: .47–.62; *p* < .0001; I^2^ = 32.22%; Supplementary Figure S3). Eight of the 16 studies reported ratios adjusted for various confounders, using multivariate analyses or by matching the PKT and nPKT group (5, 6, 8, 9, 18, 22, 25, 29). Pooling of these adjusted ratios showed a similar result (*n* = 16,715; RR: .61; CI: .40–.92; *p* = .018; I^2^ = 60.7%; Supplementary Figure S4). The adjusted ratios and confounders are presented in Supplementary Table S3.

In an attempt to explain the heterogeneity between studies for overall graft loss, a mixed-effect analysis was performed which looked at the role of four moderator variables: the percentage of HD patients in the nPKT group, length of follow-up, percentage of LD, and the year of publication (Supplementary Figures S5–S8). None of these variables were found to significantly influence the relative risk of graft loss. It may be worth noting that on visual inspection of the forest plot, the heterogeneity is in the size of effect rather than the direction of effect.

Five studies (5, 6, 23, 26, 27) reported on overall graft loss for LD, and the pooled analysis showed that PKT significantly reduced the risk of graft loss (*n* = 4,973; RR: .57; CI: .46–.69; *p* < .0001; I^2^ = 0%; Supplementary Figure S9).

Two studies (5, 23) reported on overall graft survival in DD recipients. Amaral et al (5) reported a significantly higher 5-year graft survival rate in PKT patients compared to nPKT patients (85.4% vs. 76.4%; *p* < .001). However, Vats et al (23) reported similar 3-year graft survival in PKT versus nPKT (PD and HD) patients.

Death-censored graft loss was reported in two studies (9, 30) for LD and DD data combined. Garcia et al (9) reported a higher 12-, 36-, 60- and 90-month death-censored graft survival rate, adjusted by donor type, in PKT patients compared with nPKT patients (97% vs. 87%; 92% vs. 79%; 86% vs. 72%; 76% vs. 65%, respectively). The difference was significant at 90 months (*p* < .05); however, the study did not clearly report if the differences were significant at the other time points. The study by Fitzwater et al (30), found no significant difference in the 2-year death-censored graft loss between PKT and nPKT.

### Delayed Graft Function

DGF was reported in three studies (17, 25, 27). The RR for the incidence of DGF was .57 (*n* = 4,871; CI: .22–1.50; *p* = .26; Supplementary Figure S10). Heterogeneity was high (I^2^ = 81.51%). We could not explore heterogeneity as the number of studies was too small.

DGF for LD was reported in two studies (17,27). Cransberg et al (17) showed a slightly higher incidence of DGF in PKT patients (3.5% vs. 2.4%), but did not report if this difference was significant. No significant difference was observed between PKT vs. nPKT in terms of DGF in the study by Kim et al (27).

The only study that reported on DGF in DD patients was Cransberg et al (17), which observed no difference in the DGF rate between PKT and nPKT.

### Acute Rejection

**FIGURE 4:**
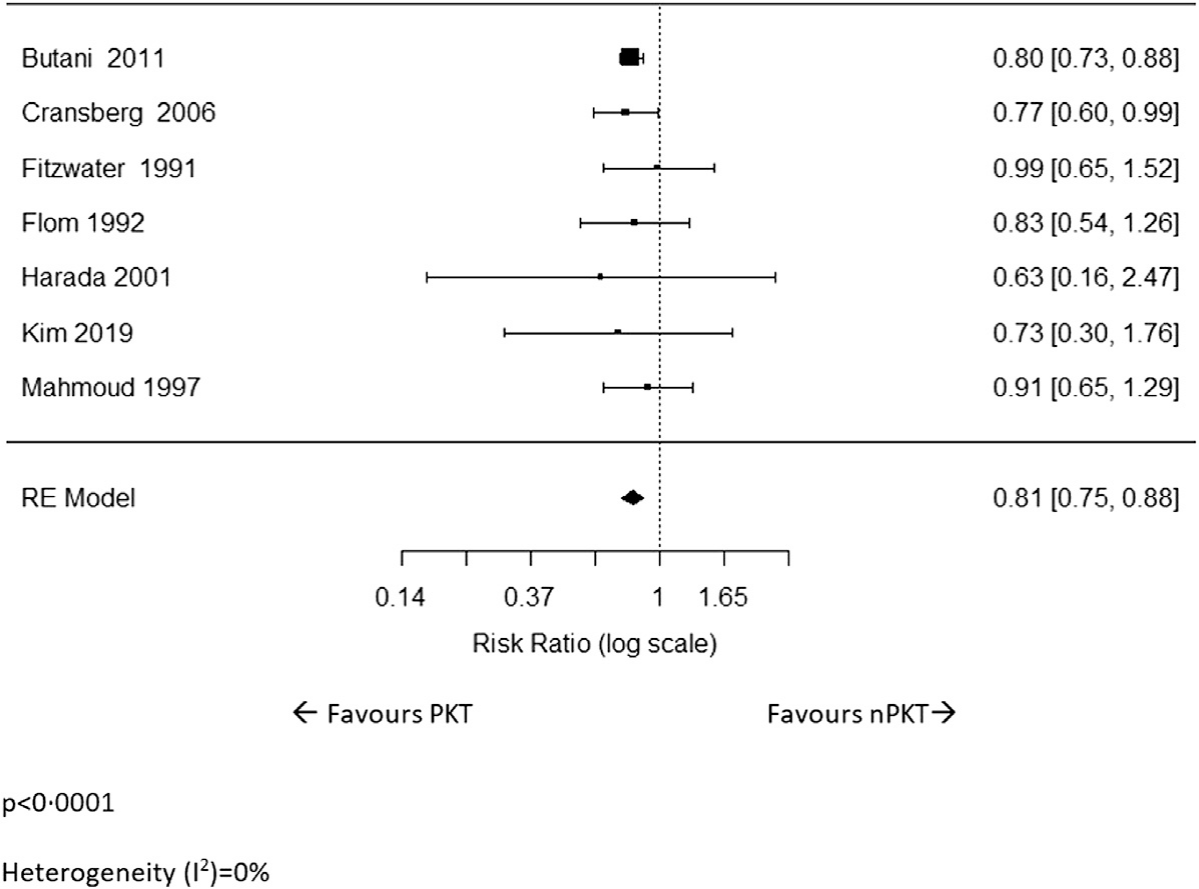
The relative risk of acute rejection for PKT vs. nPKT.

Incidence of acute rejection was reported in seven studies (6, 17, 22, 25–27, 30). The pooled analysis revealed that the risk of acute rejection in PKT patients was significantly lower than that of nPKT patients (*n* = 4,897; RR: .81; CI: .75–.88; *p* < .0001; Figure 4). Heterogeneity was low (I^2^ = 0%). Similar results were observed after excluding Fitzwater et al (30) from the analysis due to overlapping country and study period (*n* = 4,851; RR: .81; CI: .74–.87; *p* < .0001; I^2^ = 0%; Supplementary Figure S11). Of the seven studies, only two (6, 22) adjusted for confounders; hence, a pooled estimate of the adjusted acute rejection rate could not be calculated.

Three studies (6, 26, 27) reported on the rate of acute rejection for LD. Although the effect size was similar to the overall analysis, it did not reach statistical significance (*n* = 247; RR: .79; CI: .55–1.15; *p* = .22; I^2^ = 0%; Supplementary Figure S12).

Cransberg et al (17) was the only study that included data on acute rejection for DD. In the study, a significantly higher percentage of patients remained acute rejection-free following PKT than after nPKT (52% vs. 37%; *p* = .039) at 3 years.

### Cardiovascular Morbidity, Infections and Malignancy

Two studies reported cardiovascular morbidity outcomes (17, 31). Cransberg et al (17) measured the incidence of severe hypertension between PKT vs. nPKT at one, three and 5 years post-transplant, and found significantly lower incidence of severe hypertension in the PKT group in the third year (40% vs. 64%; *p* = .016), among patients with DD transplants. The study by Sinha and Marks (31) also showed a significantly lower incidence of hypertension in the PKT versus nPKT group (31% vs. 53%; *p* = .02) for combined LD and DD data. No studies reported on infections and malignancy.

### Renal Function

Renal function was reported in six studies as either eGFR or serum creatinine, with four studies (20, 22, 30, 32) reporting on LD and DD data combined. Mahmoud et al (22) evaluated the mean GFR at one and 4 years post-transplant, and found no statistical differences in the GFR values between the PKT and nPKT group at both follow-ups. The study by Kaya et al (20) also showed no significant difference in the mean GFR between these groups within a median follow-up of 7.23 years. Duzova et al (32) measured the mean GFR values at one, two, three and 5 years after transplantation, and reported a significantly lower mean GFR in the PKT group only in the third year (mean ± standard deviation (SD): 86 ± 31 ml/min/m^2^ vs. 101 ± 31 ml/min/m^2^; *p* < .05). Likewise, Fitzwater et al (30) reported no statistical differences in the serum creatinine levels between PKT and nPKT at 1 month, 3 months, 6 months, 1 year and 2 years post-transplant.

Two studies (26, 27) reported renal function for LD only. Kim et al (27) reported no differences between PKT and nPKT in the median GFR at 1 month and 1 year. Flom et al (26) reported a higher mean (±SD) GFR for PKT (68 ± 28 ml/min/1.73 m^2^) versus nPKT (HD and PD) (both 60 ± 26 ml/min/1.73 m^2^), calculated over a median follow-up of 3.5, 3.6 and 5.1 years for PKT, PD and HD respectively. However, the study did not report whether this difference was significant.

### Primary Non-Function

No studies reported on primary-non function.

### Quality of Life

Quality of life was reported in only two studies (6, 33). Splinter et al (33) assessed the health-related quality of life (HRQoL) of patients who spent at least 6 months on their treatment modality, using the PedsQL™ questionnaire. The PedsQL™ consisted of five major domains, including physical health, emotional functioning, social functioning, school functioning, and psychosocial health. The mean ± SD HRQoL scores for physical health was significantly higher in the PKT vs. nPKT group (78.6 ± 18.0 vs. 70.4 ± 20.5; *p* < .05), but showed no differences between the groups for the other domains. Harada et al (6) asked patients about the benefits and disadvantages of renal transplantation. The percentage of patients that reported feeling satisfied with the improvement in their physical condition was significantly higher in the PKT vs. the nPKT group (*p* < .01). On the other hand, a significantly higher percentage of patients in the nPKT group reported satisfaction related to the freedom from restrictions of liquid intake, daily diet and time spent on dialysis, following renal transplantation (*p* < .01). No significant differences were observed between the two groups regarding disadvantages felt due to renal transplantation, which included anxiety about the fate of the renal graft and annoyance resulting from frequent hospital visits and daily medications.

### Return to School

No studies reported data on return to school.

### Height/Growth

Three studies (6, 8, 31) reported findings on the height/growth of patients. Harada et al (6) assessed the mean ± SD heights of the patients at transplantation and at one and 3 years post-transplant, using the national cross-sectional standard growth chart for boys and girls. The study showed significantly better mean ± SD height in the PKT vs. nPKT group at transplantation (−.84 ± 0.73 vs. −2.86 ± 1.93; *p* < .05) and at 3 years post-transplant (−.53 ± 1.65 vs. −3.22 ± 1.94; *p* < 0.05), only for patients less than 15 years old. Sinha and Marks (31), who measured the height of the patients at the last clinical visit (range 1–15 years) using the median standard deviation score (SDS), found no significant differences in the scores between the two groups. Similar results were reported by Offner et al (8), who also used the median SDS to measure the height of the patients at 1 year post-transplantation.

### Primary Kidney Transplant

Secondary analyses comparing PKT versus nPKT patients with primary KT are presented in Supplementary Figures S13–S15.

## Discussion

The available evidence from observational studies suggests that PKT significantly lowers the risk of graft loss and acute rejection compared to nPKT. PKT patients with LD transplants are seen to benefit from a reduced risk of patient death as well as overall graft loss. Most studies in our review showed nonsignificant differences in post-transplant renal function between PKT and nPKT patients. Regarding other outcomes, such as cardiovascular morbidity, quality of life and height/growth, it was not possible to draw firm conclusions due to the limited evidence available. However, with regard to quality of life, patients reported improvement in physical condition better in the PKT than the nPKT group. There were not enough data to draw firm conclusions regarding different outcomes for DD and LD kidney transplantation.

Our results agree with the findings of the systematic review by Abramowicz et al (34), which looked at a combination of paediatric and adult KT recipients and suggested PKT offers better allograft survival. The same benefit has been observed in studies performed on adult PKT patients (35, 36). Research explaining the reasons for this benefit, especially specific to paediatric patients, is scarce. It is possible that several confounding factors have accounted for some or all of this observed survival advantage. Studies have shown that rates of PKT are significantly higher in children who are white versus other races, and males versus females (37–39). This may result in selection bias, which in turn may affect graft survival.

We attempt to explain the association between PKT and higher graft survival by analysing data in adult studies because of the lack of data on paediatric patients. It should, however, be noted that it remains unclear to what extent these adult data can be applied to the paediatric patients. Firstly, some authors have speculated that the association of between PKT and a reduced risk of graft loss may have been influenced by higher residual renal function of native kidney observed in PKT patients at transplantation, compared to nPKT patients. However, three studies have found that PKT with higher pre-transplant eGFR is not linked to better graft survival (40–42), suggesting that pre-transplant residual renal function may not be one of the major factors affecting graft survival. Secondly, the survival benefit of PKT may be due to the avoidance of comorbidities, such as cardiovascular disease, that are associated with dialysis (43). A study by Prezelin-Reydit et al (44), however, found that the adjusting for cardiovascular comorbidities and diabetes mellitus did not alter the link between PKT and the reduction in the hazard of graft failure. This agrees with our subgroup analysis of adjusted risks, which still showed a graft survival advantage for PKT. Lastly, as PKT take place earlier in a patient’s natural history of disease compared to nPKT, there are concerns that this “lead time” may bias observational studies to favour PKT as the optimal treatment modality (11, 45). However, Gill et al (36) demonstrated that PKT and nPKT patients with at least 2 years of allograft survival established similar baseline GFR levels at 6 months post-transplant, disapproving the hypothesis that the graft survival benefit linked to PKT may be a consequence of lead time bias due to earlier transplantation of PKT patients with preserved native kidney function.

Another significant finding in our meta-analysis is a lower incidence of acute rejection in PKT patients which may be explained by the biological differences observed in the immune reactivity of PKT versus nPKT patients (11). These differences are not yet well understood and are somewhat counterintuitive; therefore, further in-depth immunological studies into T cell senescence and allo-immunity in both groups are warranted.

This study had several weaknesses. It only included observational studies, which by nature are frequently subject to confounding and bias, which may lead to false-positive findings (46). Additionally, although current paediatric kidney transplantation guidance advises PKT whenever possible, in reality, some non-adherent children may be initiated on dialysis before receiving a transplant. This practice introduces a bias and it may be an additional unaccounted confounder in our results. The small number of studies in some of the pooled analyses preclude finding convincing evidence for the outcomes, for example for delayed graft function. Heterogeneity was high for some of the outcomes, and could not always be explored due to the small number of studies. Definitions of reported outcomes were not clearly stated for some studies, e.g., overall graft survival or death-censored graft survival. We were unable to perform separate analyses for LD versus DD patients for most outcomes due to limited number of studies that presented these data separately. It was also unclear from some of the included studies whether there were any pre-emptive second transplants included in the study populations. Although we attempted to address the possible role of confounding variables, such as socio-economic status, health literacy, psychosocial support, lead time bias and recurrence of primary ESKD, on overall graft survival by pooling adjusted ratios, this is limited to the adjustments used in the original analyses and additional confounders may still be present. Another limitation is the inconsistent reporting of dialysis vintage, making it difficult to assess the impact of different durations of dialysis on transplant outcomes.

Our systematic review also highlights the inconsistent and poor reporting of certain outcomes that are relevant to paediatric ESKD patients, such as cardiovascular disease and quality of life. Studies have shown that absence from school, social engagement, symptoms (feeling ill or pain), hospitalisation, poor sleep and fatigue are important to children with ESKD (47–49), however, these outcomes were poorly reported or not reported at all by the studies included in the review. Future studies should report the core outcomes established by the SONG-Kids initiative (50) to ensure that outcomes relevant to children are included in research proposals.

In conclusion, systematic review of observational studies showed that paediatric PKT patients have a lower risk of overall graft loss and acute rejection than nPKT patients. While no difference was seen in overall patient mortality, PKT appeared to significantly lower the risk of patient death in LD patients. Therefore, it is important to develop pathways that ensure PKT options for as many paediatric ESKD patients as possible, especially emphasising on living donation. With education of paediatric patients and carers early in the disease process about LD PKT, a timely transplant or timely waitlisting for DD KT (in absence of LD options) can be achieved for many patients. This also calls for a redesign of the default renal replacement therapy pathway, which unfortunately is still set to dialysis before transplantation.

## Author's Note

The abstract of the paper received the 2021 Marius Renard Award for Best Abstract on Paediatric Transplantation (a joint ESOT and IPTA (International Pediatric Transplant Association) award) during the 2021 ESOT congress.

## Abbreviations

CI, confidence interval; DD, deceased donor; DGF, delayed graft function; eGFR, estimated glomerular filtration rate; ESKD, end-stage kidney disease; HD, haemodialysis; HRQoL, health-related quality of life; KT, kidney transplantation; LD, living donor; nPKT, non-preemptive kidney transplantation; PD, peritoneal dialysis; PKT, preemptive kidney transplantation; RR, relative risk; SD, standard deviation; SDS, standard deviation score.
