# Supplementary material for: Is Preemptive Kidney Transplantation Associated With Improved Outcomes when Compared to Non-preemptive Kidney Transplantation in Children? A Systematic Review and Meta-Analysis
Source: Transpl Int. 2022 Mar 17;35:10315. doi: 10.3389/ti.2022.10315 (PMC8967954; doi:10.3389/ti.2022.10315)
Supplement: Supplementary file 1 [file DataSheet1.docx]

**SUPPLEMENTAL DIGITAL CONTENT**

Figure S1 Forest plot showing the relative risk of patient death for PKT versus nPKT excluding overlapping studies.


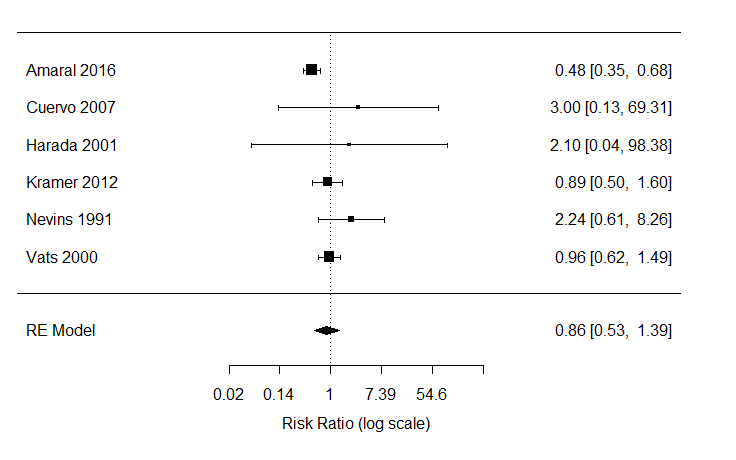


🡨 Favours PKT Favours nPKT🡪

Figure S2 Forest plot showing the relative risk of patient death for PKT versus nPKT for LD transplants.


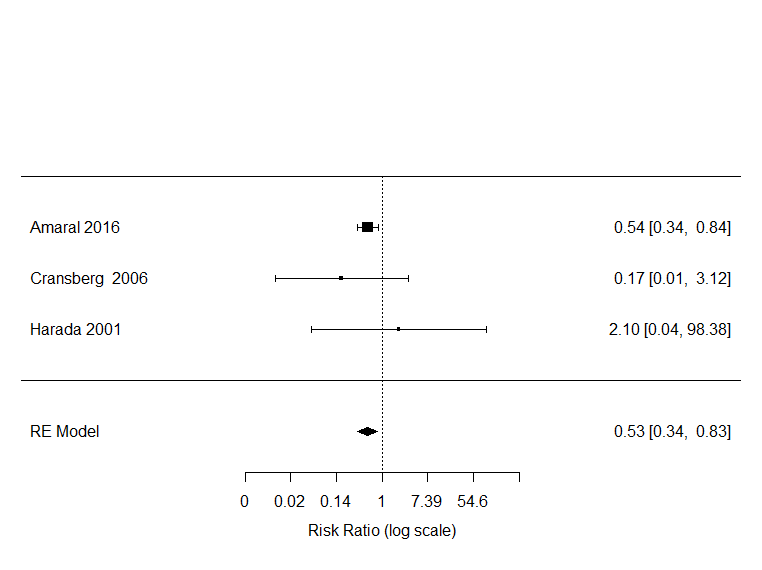


🡨 Favours PKT Favours nPKT🡪

Figure S3 Forest plot showing the relative risk of overall graft loss for PKT versus nPKT excluding overlapping studies.


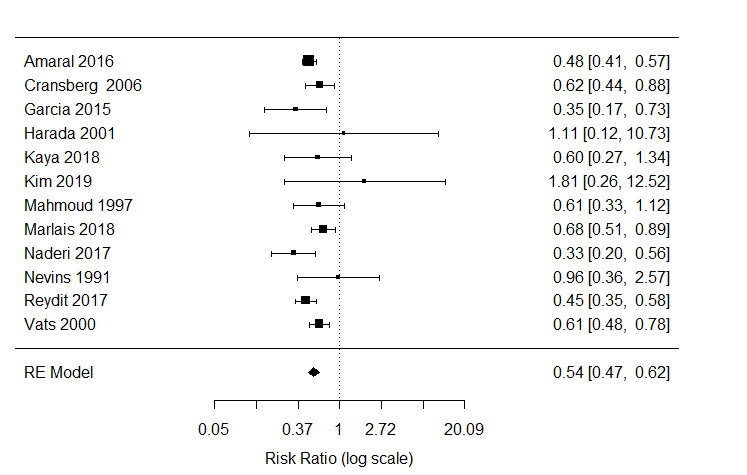


🡨 Favours PKT Favours nPKT🡪

Figure S4 Forest plot showing the relative risk of overall graft loss for PKT versus nPKT adjusted for confounders.

🡨 Favours PKT Favours nPKT🡪


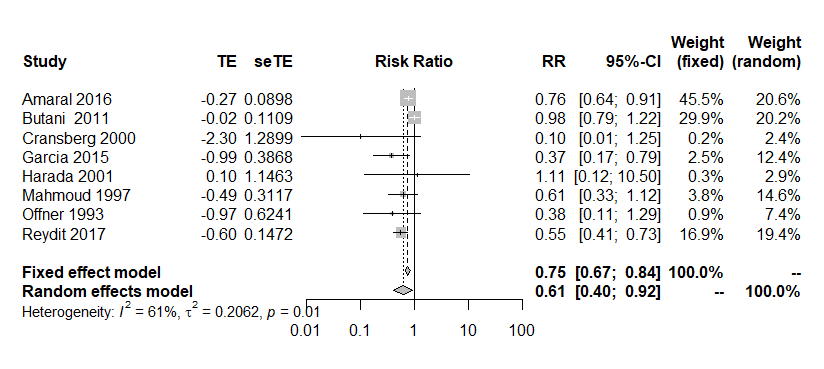


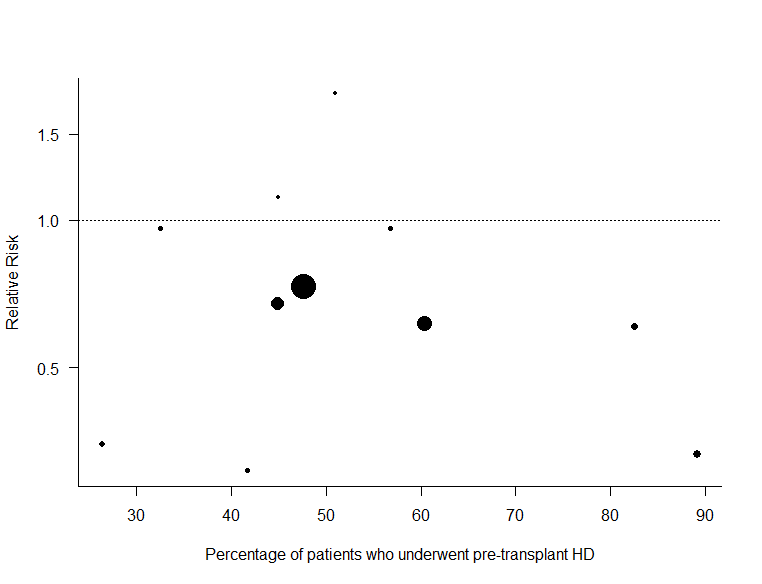
**Figure S5** Relative risk plotted against the percentage of HD patients in the nPKT group in each study.

Moderator variable: Percentage of HD in the nPKT group

Number of studies: 11

I^2^ (residual heterogeneity / unaccounted variability): 6.21%

H^2^ (unaccounted variability / sampling variability): 1.07

R^2^ (amount of heterogeneity accounted for): 89.79%

Test for Residual Heterogeneity: QE(df = 9) = 12.8641, P= 0.1689

Test of Moderators (coefficient 2): QM(df = 1) = 2.8947, P= 0.0889

**Figure S6** Relative risk plotted against the final follow-up duration in each study
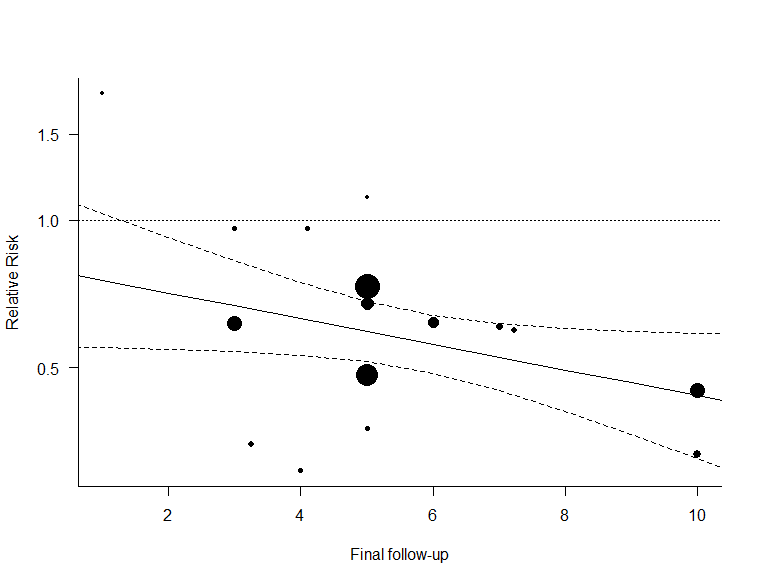
.

Moderator variable: Final follow-up

Number of studies: 16

I^2^ (residual heterogeneity / unaccounted variability): 41.38%

H^2^ (unaccounted variability / sampling variability): 1.71

R^2^ (amount of heterogeneity accounted for): 29.16%

Test for Residual Heterogeneity: QE(df = 14) = 25.9458, P= 0.0263

Test of Moderators (coefficient 2): QM(df = 1) = 3.7810, P= 0.0518


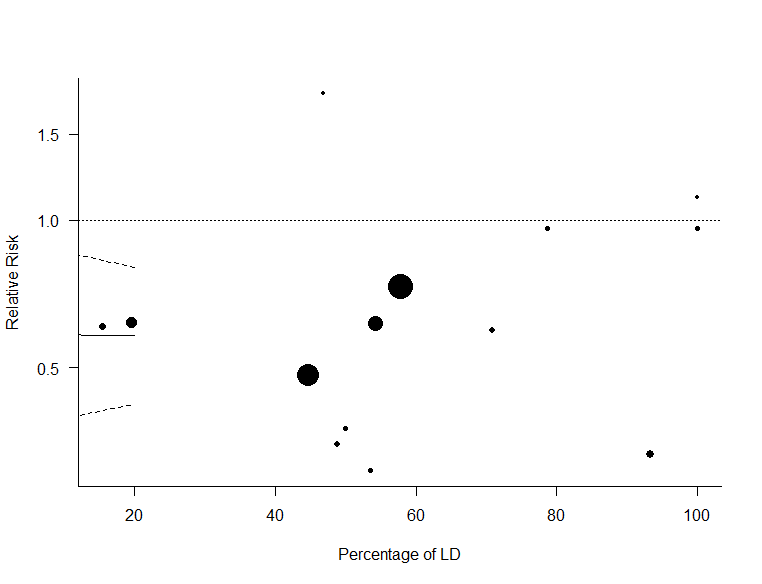
**Figure S7** Relative risk plotted against the percentage of LD in each study.

Moderator variable: Percentage of LD

Number of studies: 14

I^2^ (residual heterogeneity / unaccounted variability): 50.95%

H^2^ (unaccounted variability / sampling variability): 2.04

R^2^ (amount of heterogeneity accounted for): 0.00%

Test for residual heterogeneity: QE(df = 12) = 27.1443, P= 0.0074

Test of Moderators (coefficient 2): QM(df = 1) = 0.0179, P= 0.8935

**
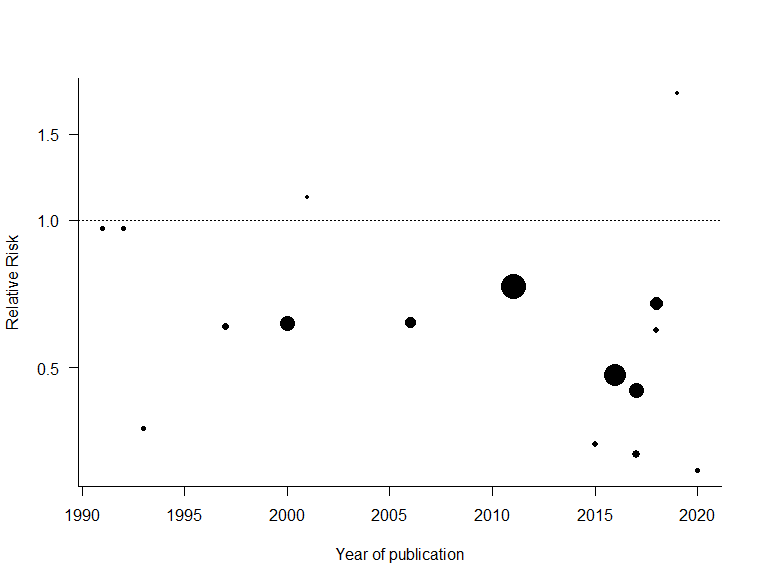
Figure S8** Relative risk plotted against the year of publication for each study.

Moderator variable: Year of publication

Number of studies: 16

I^2^ (residual heterogeneity / unaccounted variability): 47.09%

H^2^ (unaccounted variability / sampling variability): 1.89

R^2^ (amount of heterogeneity accounted for): 11.16%

Test for Residual Heterogeneity: QE(df = 14) = 27.6813, P= 0.0157

Test of Moderators (coefficient 2): QM(df = 1) = 2.5440, P= 0.1107

Figure S9 Forest plot showing the relative risk of graft loss for PKT versus nPKT for LD transplants.

🡨 Favours PKT Favours nPKT🡪


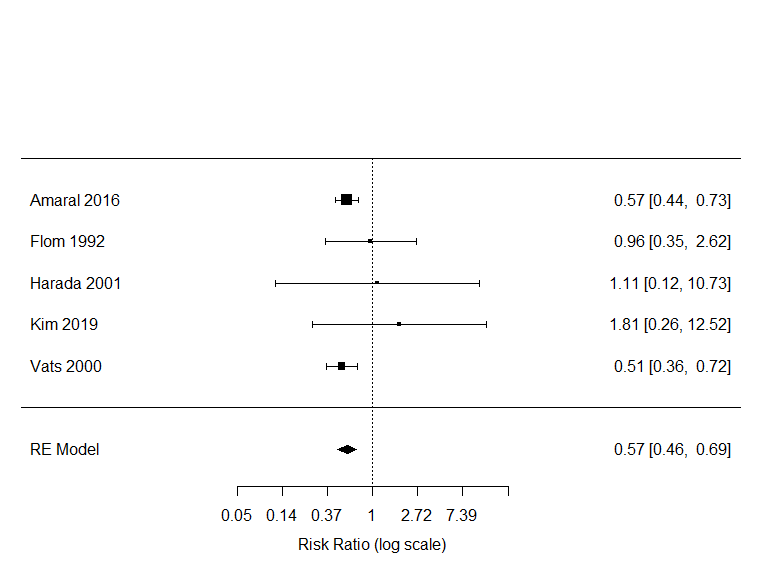


*]*

**Figure 8.** Forest plot showing the relative risk of overall graft loss for PKT versus nPKT for LD.

Figure S10 Forest plot showing the relative risk of delayed graft function for PKT versus nPKT.


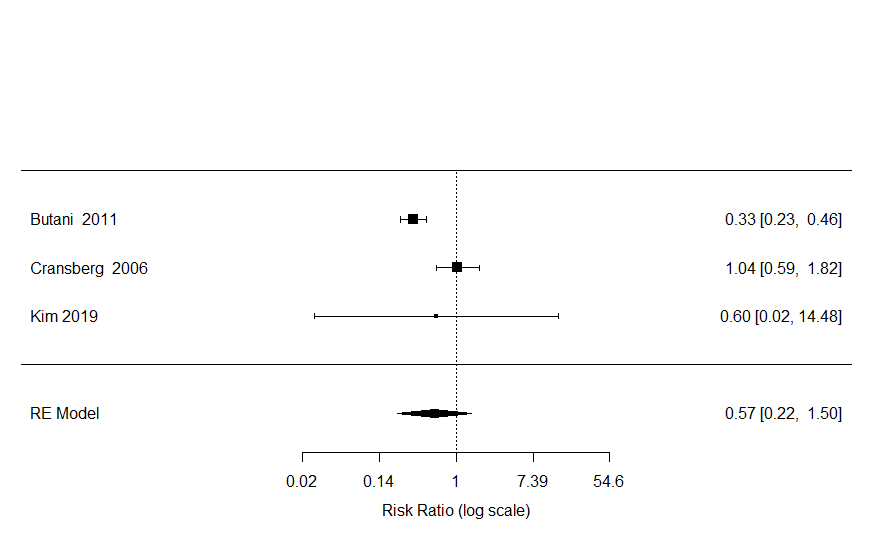


🡨 Favours PKT Favours nPKT🡪

Figure S11 Forest plot showing the relative risk of acute rejection for PKT versus nPKT excluding overlapping studies.


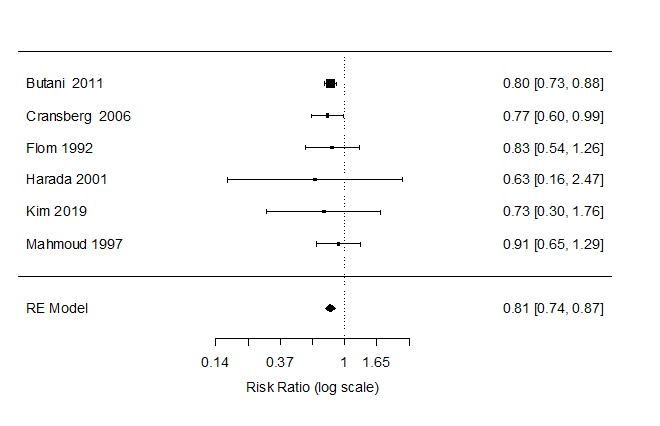


🡨 Favours PKT Favours nPKT🡪

Figure S12 Forest plot showing the relative risk of acute rejection for PKT versus nPKT for LD.


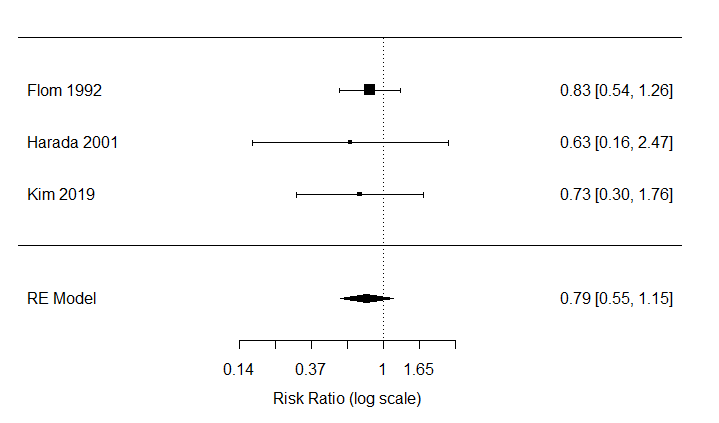


🡨 Favours PKT Favours nPKT🡪

Figure S13 Forest plot showing the relative risk of patient death for PKT versus nPKT for patients with a primary kidney transplant.


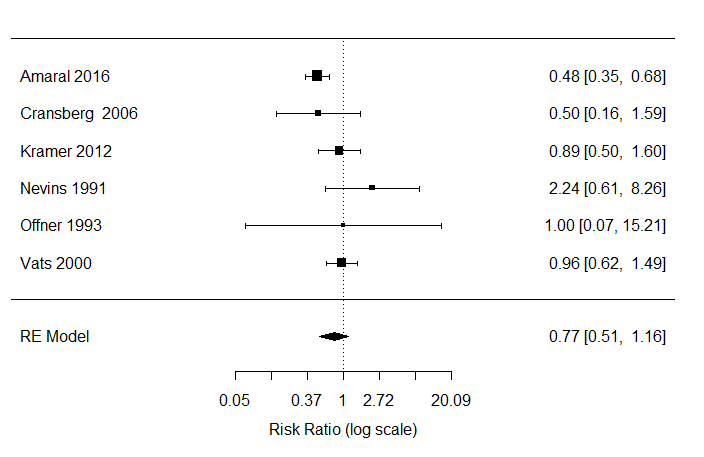


🡨 Favours PKT Favours nPKT🡪

p=0.21

Heterogeneity (I^2^)=51.50%

Figure S14 Forest plot showing the relative risk of overall graft loss for PKT versus nPKT for patients with a primary kidney transplant.


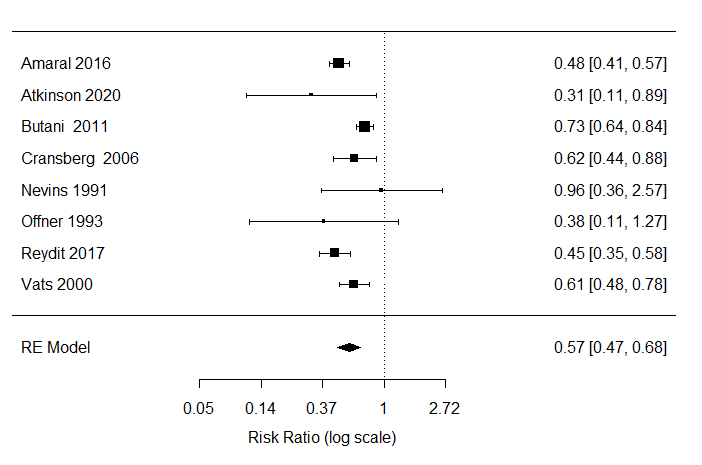


🡨 Favours PKT Favours nPKT🡪

p<0.0001

Heterogeneity (I^2^)=64.94%

Figure S15 Forest plot showing the relative risk of acute rejection for PKT versus nPKT for patients with a primary kidney transplant.


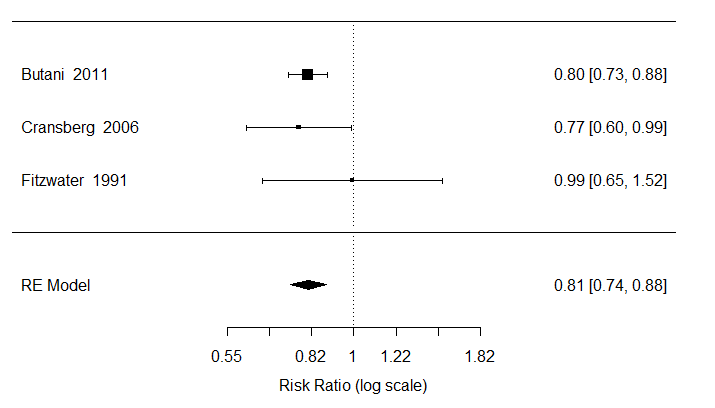


🡨 Favours PKT Favours nPKT🡪

p<0.0001

Heterogeneity (I^2^)=0.00%

**Table S1.** Search strategy

**Date of search:** 2020/07/31

| **Database** | **Search terms** | **With Duplicates** | **Without**  **Duplicates** |
| --- | --- | --- | --- |
| Embase.com | ('kidney transplantation'/exp OR (kidney/de AND transplantation/de) OR (((kidney* OR renal) NEAR/3 (transplant* OR homotransplant* OR autotransplant* OR allograft* OR allotransplant* OR graft* OR recipient*))):ab,ti) AND (preemptiv* OR (pre NEXT/1 emptiv*) OR (('prior to' OR before OR pre) NEXT/3 ('end stage' OR dialys* OR esrd))):ab,ti | 3500 | 3470 |
| Medline (OvidSP) | ("kidney transplantation"/ OR (kidney/ AND (transplantation/ OR transplantation.xs.)) OR (((kidney* OR renal) ADJ3 (transplant* OR homotransplant* OR autotransplant* OR allograft* OR allotransplant* OR graft* OR recipient*))).ab,ti.) AND (preemptiv* OR (pre ADJ emptiv*) OR (("prior to" OR before OR pre) ADJ3 ("end stage" OR dialys* OR esrd))).ab,ti. | 2146 | 617 |
| Web-of-science | TS=(((((kidney* OR renal) NEAR/3 (transplant* OR homotransplant* OR autotransplant* OR allograft* OR allotransplant* OR graft* OR recipient*)))) AND (preemptiv* OR (pre NEAR/1 emptiv*) OR (("prior to" OR before OR pre) NEAR/3 ("end stage" OR dialys* OR esrd)))) | 2348 | 562 |
| Cochrane | ((((kidney* OR renal) NEAR/3 (transplant* OR homotransplant* OR autotransplant* OR allograft* OR allotransplant* OR graft* OR recipient*))):ab,ti) AND (preemptiv* OR (pre NEXT/1 emptiv*) OR (('prior to' OR before OR pre) NEXT/3 ('end stage' OR dialys* OR esrd))):ab,ti | 389 | 281 |
| Google Scholar | "kidney\|renal transplantation\|homotransplantation\|autotransplantation\|allograft\|allotransplantation\|graft\|recipients" preemptive\|"pre emptive"\|"prior-to\|before\|pre end-stage\|dialysis\|esrd" | 200 | 55 |
| **Total** |  | **8583** | **4985** |

**Table S2.** Methodological quality scores on the items of the Downs and Black checklist

|  | Amaral | Atkinson | Butani | Cransberg | Fitzwater | Flom | Garcia | Harada | Kim | Kramer | Mahmoud | Naderi | Nevins | Offner | Sinha | Splinter | Vats |
| --- | --- | --- | --- | --- | --- | --- | --- | --- | --- | --- | --- | --- | --- | --- | --- | --- | --- |
| ***Reporting*** | | | | | | | | | | | | | | | | | |
| 1. Is the hypothesis/aim/objective of the study clearly described? | 1 | 1 | 1 | 1 | 1 | 1 | 1 | 1 | 1 | 1 | 1 | 1 | 1 | 1 | 1 | 1 | 1 |
| 2. Are the main outcomes to be measured clearly described in the Introduction or Methods section? | 1 | 1 | 1 | 1 | 1 | 1 | 1 | 1 | 1 | 1 | 1 | 1 | 0 | 1 | 1 | 1 | 1 |
| 3. Are the characteristics of the patients included in the study clearly described? | 1 | 1 | 1 | 1 | 1 | 1 | 0 | 1 | 1 | 1 | 0 | 1 | 1 | 0 | 1 | 1 | 1 |
| 4. Are the interventions of interest clearly described? | 1 | 1 | 1 | 1 | 1 | 1 | 1 | 1 | 1 | 1 | 1 | 1 | 1 | 1 | 1 | 1 | 1 |
| 5. Are the distributions of principal confounders (recipient age, underlying renal disease, LD versus DD, previous transplant history, measure of sensitization e.g. PRA and HLA mismatch, and ethnicity) in each group of subjects to be compared clearly described?* | 2 | 1 | 1 | 1 | 0 | 1 | 1 | 1 | 1 | 0 | 1 | 0 | 0 | 1 | 2 | 0 | 2 |
| 6. Are the main findings of the study clearly described? | 1 | 1 | 0 | 1 | 1 | 1 | 1 | 1 | 1 | 1 | 1 | 1 | 0 | 1 | 1 | 1 | 1 |
| 7. Does the study provide estimates of the random variability in the data for the main outcomes? | 1 | 0 | 0 | 0 | 1 | 0 | 1 | 0 | 1 | 1 | 1 | 1 | 0 | 0 | 1 | 1 | 0 |
| 8. Have all important adverse events that may be a consequence of the intervention been reported? | 0 | 0 | 0 | 0 | 0 | 0 | 0 | 0 | 0 | 0 | 1 | 0 | 0 | 0 | 0 | 0 | 0 |
| 9. Have the characteristics of patients lost to follow‐up been described? | 0 | 0 | 0 | 0 | 0 | 0 | 0 | 0 | 0 | 0 | 0 | 0 | 0 | 0 | 0 | NA | 0 |
| 10. Have actual probability values been reported? | 1 | 1 | 1 | 1 | 0 | 0 | 0 | 0 | 1 | 0 | 0 | 1 | 1 | 0 | 1 | 0 | 1 |
| ***External validity*** | | | | | | | | | | | | | | | | | |
| 11. Were the subjects asked to participate in the study representative of the entire population from which they were recruited? | 1 | 1 | 1 | 1 | 0 | 1 | 1 | 1 | 1 | 1 | 1 | 1 | 0 | 0 | 1 | 1 | 1 |
| 12. Were those subjects who were prepared to participate representative of the entire population from which they were recruited? | 1 | 1 | 1 | 1 | 1 | 1 | 1 | 1 | 1 | 1 | 1 | 1 | 0 | 0 | 1 | 1 | 1 |
| 13. Were the staff, places, and facilities where the patients were treated, representative of the treatment the majority of patients receive? | 1 | 1 | 1 | 1 | 1 | 1 | 1 | 1 | 1 | 1 | 1 | 1 | 1 | 1 | 1 | 1 | 1 |
| ***Internal validity (bias)*** | | | | | | | | | | | | | | | | | |
| 14. Was an attempt made to blind study subjects to the intervention they have received? | 0 | 0 | 0 | 0 | 0 | 0 | 0 | 0 | 0 | 0 | 0 | 0 | 0 | 0 | 0 | 0 | 0 |
| 15. Was an attempt made to blind those measuring the main outcomes of the intervention? | 0 | 0 | 0 | 0 | 0 | 0 | 0 | 0 | 0 | 0 | 0 | 0 | 0 | 0 | 0 | 0 | 0 |
| 16. If any of the results of the study were based on “data dredging”, was this made clear? | 0 | 1 | 1 | 1 | 1 | 1 | 1 | 1 | 0 | 1 | 0 | 1 | 0 | 1 | 1 | 1 | 1 |
| 17. In trials and cohort studies, do the analyses adjust for different lengths of follow‐up of patients, or in case‐control studies, is the time period between the intervention and outcome the same for cases and controls? | 1 | 1 | 1 | 1 | 1 | 1 | 1 | 0 | 1 | 1 | 1 | 1 | 1 | 1 | NA | NA | 1 |
| 18. Were the statistical tests used to assess the main outcomes appropriate? | 1 | 1 | 1 | 1 | 1 | 1 | 1 | 1 | 1 | 1 | 1 | 1 | 1 | 1 | 1 | 1 | 1 |
| 20. Were the main outcome measures used accurate (valid and reliable)? | 1 | 1 | 1 | 1 | 1 | 1 | 1 | 1 | 1 | 1 | 1 | 1 | 1 | 1 | 1 | 1 | 1 |
| ***Internal validity (confounding)*** | | | | | | | | | | | | | | | | | |
| 21. Were the patients in different intervention groups (trials and cohort studies) or were the cases and controls (case‐control studies) recruited from the same population? | 1 | 1 | 1 | 1 | 1 | 1 | 1 | 1 | 1 | 1 | 1 | 1 | 1 | 1 | 1 | 1 | 1 |
| 22. Were study subjects in different intervention groups (trials and cohort studies) or were the cases and controls (case‐control studies) recruited over the same period of time? | 1 | 1 | 1 | 1 | 1 | 1 | 1 | 1 | 1 | 1 | 1 | 1 | 1 | 1 | 1 | 1 | 1 |
| 23. Were study subjects randomised to intervention groups? | 0 | 0 | 0 | 0 | 0 | 0 | 0 | 0 | 0 | 0 | 0 | 0 | 0 | 0 | 0 | 0 | 0 |
| 24. Was the randomised intervention assignment concealed from both patients and health staff until recruitment was complete and irrevocable? | 0 | 0 | 0 | 0 | 0 | 0 | 0 | 0 | 0 | 0 | 0 | 0 | 0 | 0 | 0 | 0 | 0 |
| 25. Was there adequate adjustment for confounding in the analyses from which the main findings were drawn? | 1 | 1 | 1 | 1 | 0 | 0 | 1 | 1 | 0 | 1 | 1 | 0 | 0 | 1 | 0 | 1 | 1 |
| 26. Were losses of patients to follow‐up taken into account? | 1 | 1 | 1 | 1 | 0 | 0 | 1 | 1 | 0 | 1 | 1 | 0 | 0 | 0 | 0 | NA | 1 |
| Total score | 19 | 18 | 17 | 18 | 14 | 15 | 17 | 16 | 16 | 17 | 17 | 16 | 10 | 13 | 17 | 15 | 19 |

^1 = yes; 0 = no or unable to determine; NA = not applicable. For question 5, the possible answers were 0, 1 or 2, where 0 = described 1-2 confounders, 1 = described 3-4 confounders or 2 = described 5-6 confounders.^

**Table S3.** Adjusted confounding variables

| **Author (Year)** | **HR/RR of graft loss (PKT versus nPKT)** | **Adjusted variables** |
| --- | --- | --- |
| Amaral (2016) | HR: 0.76 (0.64-0.91) | Multivariable model adjusted for sex, aetiology of end-stage renal disease, age at time of transplantation, ethnicity/race,  panel reactive antibody, donor type (in combined donor type  models), insurance status at the time of transplantation, neighbourhood poverty,  and cold ischemia time (in deceased donor recipient models). |
| Butani (2011) | RR: 0.98 (0.79-1.22) | Multivariable model adjusted for donor variables— age, gender, ethnicity, and cause of death;  Recipient variables— age, gender, ethnicity, and cause of ESKD;  Transplant-related variables—HLA match, HLA-DR match, panel reactive antibody, cold ischemia time, transplant year (to account for changes in postoperativecare and immunosuppressive protocols), and use of induction immunosuppressive agents. |
| Cransberg (2000) | RR 0.10 (0.01-1.25)* | PKT and nPKT group matched for age, donor source and primary renal disease. |
| Garcia (2015) | HR: 0.37 (0.18-0.82) | Multivariable model adjusted for donor type and age, recipient age and sex, and primary diagnosis. |
| Harada (2001) | RR: 1.11 (0.12-10.73)* | PKT and nPKT group matched for age. |
| Mahmoud (1997) | RR: 0.61 (0.33-1.12)* | PKT and nPKT group matched for recipient and donor age and immunological risk factors. |
| Offner (1993) | RR: 0.38 (0.11-1.27)* | PKT and nPKT group matched for year of transplantation, age, donor source, immunosuppressive regimen, and original disease. |
| Reydit (2017) | HR: 0.55 (0.41-0.73) | Multivariable model adjusted for recipient sex and age, donor type (living or deceased donor), primary kidney disease, donor age, cold ischemia time, and  HLA mismatches. |

^PKT preemptive kidney transplantation; nPKT non-preemptive kidney transplantation; ESKD end-stage kidney disease; *, calculated; HLA-DR human leukocyte antigen – DR isotype; HR hazard ratio; RR relative risk.^
